# CYP1A1 rs1048943 and rs4646903 polymorphisms associated with laryngeal cancer susceptibility among Asian populations: a meta‐analysis

**DOI:** 10.1111/jcmm.12720

**Published:** 2015-11-18

**Authors:** Wei Zeng, Yanwei Li, Eryong Lu, Min Ma

**Affiliations:** ^1^ Department of Otorhinolaryngology Head and Neck SurgeryFirst Affiliated Hospital of Henan University of Science and TechnologyLuoyangChina; ^2^ Department of OphthalmologyFirst Affiliated Hospital of Henan University of Science and TechnologyLuoyangChina

**Keywords:** CYP1A1, rs1048943, rs4646903, polymorphism, laryngeal cancer, susceptibility, meta‐analysis

## Abstract

Many studies have investigated the association between *CYP1A1* rs1048943 and rs4646903 polymorphisms and laryngeal cancer risk, but their results have been inconsistent. The PubMed and CNKI were searched for case–control studies published up to 01 July 2015. Data were extracted and pooled odds ratios (OR) with 95% confidence intervals (CI) were calculated. In this meta‐analysis, we assessed 10 published studies involving comprising 748 laryngeal cancer cases and 1558 controls of the association between *CYP1A1* rs1048943 and rs4646903 polymorphisms and laryngeal cancer risk. For *CYP1A1* rs1048943 of the homozygote G/G and G allele carriers (A/G + G/G) *versus* A/A, the pooled ORs were 1.77 (95% CI = 1.28–2.81, *P* = 0.007 for heterogeneity) and 1.86 (95% CI = 1.45–2.40, *P* = 0.000 for heterogeneity). For *CYP1A1* rs4646903 of the homozygote G/G and G allele carriers (A/G + G/G) *versus* A/A, the pooled ORs were 1.53 (95% CI = 1.31–2.21, *P* = 0.012 for heterogeneity) and 1.33(95% CI = 1.04–1.71, *P* = 0.029 for heterogeneity). In the stratified analysis by ethnicity, the significantly risks were found among Asians for both the G allele carriers and homozygote G/G. However, no significant associations were found in Caucasian population all genetic models. These results from the meta‐analysis suggest that *CYP1A1* rs1048943 and rs4646903 polymorphisms contribute to risk of laryngeal cancer among Asian populations.

## Introduction

Laryngeal cancer is one of the most common malignant tumours among head and neck cancers 1. It is considered as a complex disease resulting from the interaction between genetic background and environmental factors 1, 2. A growing body of epidemiological evidence suggests that cigarette smoking and alcohol consumption are probable important aetiological factors increasing the risk of developing laryngeal carcinoma 3. Besides, it is believed that environmental chemical pollutions, widely spread carcinogens, are difficult to be degraded in the environment and thus may have a long‐term effect on human health. Accumulating evidence indicates that genetic polymorphisms have also been extensively investigated to identify inherited genetic risk for laryngeal Squamous cell carcinoma (SCC) 4.

Cytochromes P450 (CYP450s) are haeme‐containing enzymes important to phase I‐dependent metabolism of drugs and other xenobiotics 5. Studies indicate that CYP enzymes participate in cellular functions such as the metabolism of eicosanoids, the biosynthesis of cholesterol and bile acids, synthesis and metabolism of steroids and vitamin D3, synthesis and degradation of biogenic amines, and the hydroxylation of retinoic acid and presumably other morphogens. However, the functions of several CYP enzymes remain unknown 6, 7. Many important single nucleotide polymorphisms (SNPs) have been identified in CYP genes, and such polymorphisms within these genes may play an important role in determining individual susceptibility to many cancers. Two functional SNPs in *CYP1A1* [*CYP1A1* Ile462Val (CYP1A1*2B and *2C; rs1048943) and MspI (3798 T > C; CYP1A1*2A and *2B; rs4646903)] have been identified. *CYP1A1* rs1048943 and rs4646903 polymorphisms have been extensively studied in many cancer sites among different populations, with the inconsistent and inconclusive results.

Several studies have investigated associations between *CYP1A1* rs1048943 and rs4646903 polymorphisms and laryngeal cancer risk, but there is the perception that the findings have been inconsistent. A single study may be too underpowered to detect a possible small effect of the polymorphisms on laryngeal cancer, especially when the sample size is relatively small. Different types of study populations may also contribute the disparate findings. Hence, we performed a meta‐analysis of all eligible studies to derive a more precise estimation of the associations of *CYP1A1* rs1048943 and rs4646903 polymorphisms with laryngeal cancer.

## Materials and methods

### Publication search

The electronic databases PubMed and CNKI were searched for studies to include in the present meta‐analysis, using the terms: ‘*CYP1A1*’ or ‘rs1048943’ or ‘rs4646903’, ‘polymorphism’ and ‘laryngeal cancer’. An upper date limit of 01 July 2015 was applied; no lower date limit was used. The search was performed without any restrictions on language and was focused on studies that had been conducted in humans. Concurrently, the reference lists of reviews and retrieved articles were searched manually. Only full‐text articles were included. Disagreement was resolved by discussion between the two authors. When the same patient population appeared in several publications, only the most recent or complete study was included in this meta‐analysis.

### Inclusion criteria

The included studies have to meet the following criteria: (*i*) evaluating the *CYP1A1* rs1048943 and/or rs4646903 polymorphism and laryngeal cancer risk; (*ii*) case–control studies; and (*iii*) supply the number of individual genotypes for *CYP1A1* rs1048943 and/or rs4646903 genotype in laryngeal cancer cases and controls respectively.

### Data extraction

Information was carefully extracted from all eligible publications independently by two authors according to the inclusion criteria listed above. Disagreement was resolved by discussion between the two authors.

The following data were collected from each study: first author's surname, year of publication, ethnicity, total numbers of cases and controls, and numbers of cases and controls with the AA, AG and GG genotypes respectively. Different ethnicity descents were categorized as Asian and Caucasian.

### Statistical analysis

Odds ratio (OR) with 95% confidence intervals (CI) was used to assess the strength of association between the *CYP1A1* rs1048943 and/or rs4646903 and laryngeal cancer risk. The pooled ORs for the risk associated with the genotypes of homozygote G/G and G allele carriers (A/G + G/G) with the A/A genotype were calculated. Heterogeneity assumption was checked by the chi‐square‐based *Q*‐test 8. A *P*‐value greater than 0.10 for the *Q*‐test indicates a lack of heterogeneity among studies, so the pooled OR estimate of the each study was calculated by the fixed‐effects model (the Mantel–Haenszel method) 9. Otherwise, the random‐effects model (the DerSimonian and Laird method) was used 10. One‐way sensitivity analyses were performed to assess the stability of the results, namely, a single study in the meta‐analysis was deleted each time to reflect the influence of the individual data set to the pooled OR 11. An estimate of potential publication bias was carried out by the funnel plot, in which the standard error of log (OR) of each study was plotted against its log (OR). An asymmetric plot suggests a possible publication bias. Funnel plot asymmetry was assessed by the method of Egger's linear regression test, a linear regression approach to measure the funnel plot asymmetry on the natural logarithm scale of the OR. The significance of the intercept was determined by the *t*‐test suggested by Egger (*P* < 0.05 was considered representative of statistically significant publication bias) 12. All of the calculations were performed with STATA version 11.0 (Stata Corporation, College Station, TX, USA).

## Results

### Study characteristics

**Figure 1:**
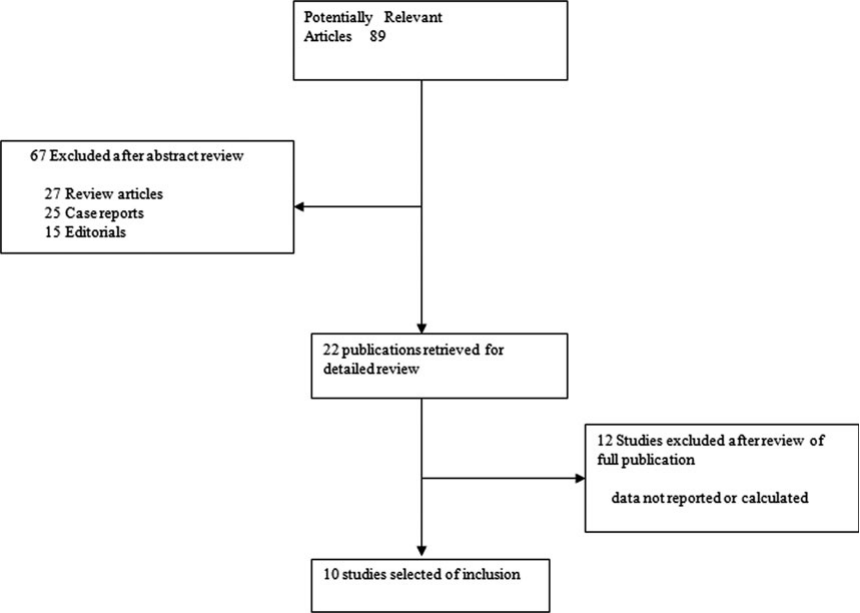
The flow diagram explaining the selection of included publications in the meta‐analysis.

The flow diagram was illustrated graphically in Figure 1. A total of 10 publications involving 748 laryngeal cancer cases and 1558 controls met the inclusion criteria and were ultimately analysed 13, 14, 15, 16, 17, 18, 19, 20, 21, 22. Table 1 presents the main characteristics of these studies. Among the 10 publications, eight were published in English, the remaining two were publish in Chinese. The sample sizes ranged from 46 to 466. Almost all of the cases were histologically confirmed. Controls were mainly healthy populations. Six studies were hospital‐based case–control studies and four were population‐based case–control studies.

**Table 1:** Distribution of CYP1A1 rs1048943 among laryngeal cancer cases and controls included in this meta‐analysis

First author‐year	Ethnicity (country of origin)	Total sample (case/control)	Source of control	Laryngeal cancer			Controls		
				AA	AG	GG	AA	AG	GG
Lei‐2002	Asian (China)	62/56	HB	17	36	9	30	22	4
Varzim‐2003	Caucasian (Portugal)	88/178	PB	66	19	3	135	43	0
Gajecka‐2005	Caucasian (Poland)	206/260	HB	191	15^a^		230	30^a^	
Sam‐2008	Asian (India)	80/220	HB	24	44	12	180	36	4
Sharma‐2010	Asian (India)	46/201	HB	35	10	1	156	34	11
Lourenco‐2011	Mixed (Brazil)	37/142	HB	25	12^a^		104	38^a^	

a The number of the combined AG and GG genotypes.

PB: population based; HB: hospital based. AA indicates wild‐type, AG indicates heterozygote, GG indicates variant homozygote.

### Meta‐analysis results

**Figure 2:**
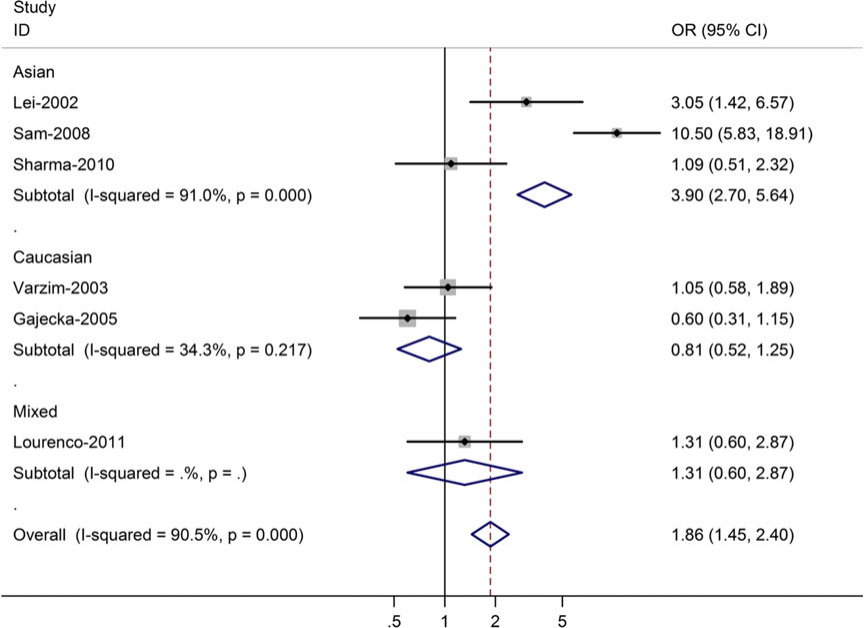
Forest plot (random‐effects model) of laryngeal cancer risk associated with CYP1A1 rs1048943 polymorphism for (AG + GG) *versus* AA. Each box represents the OR point estimate, and its area is proportional to the weight of the study. The diamond (and broken line) represents the overall summary estimate, with CI represented by its width. The unbroken vertical line is set at the null value (OR = 1.0).

The association between *CYP1A1* rs1048943 and laryngeal cancer risk was performed by meta‐analysis. Overall, for the homozygote G/G and G allele carriers (A/G + G/G), the pooled ORs for six studies were 1.77 (95% CI = 1.28–2.81, *P* = 0.007 for heterogeneity) and 1.86 (95% CI = 1.45–2.40, *P* = 0.000 for heterogeneity) (Fig. 2), when compared with the homozygous wild‐type genotype (A/A). In the stratified analysis by ethnicity, significant risks were found among Asians for both the G allele carriers (OR = 3.90, 95% CI = 2.70–5.64; *P* = 0.000 for heterogeneity) and homozygote G/G (OR = 3.29; 95% CI = 1.88–4.49; *P* = 0.006 for heterogeneity). Among Caucasian populations, no significant association was found in G/G *versus* A/A (OR = 0.93; 95% CI = 0.49–1.39), and G allele carriers *versus* A/A (OR = 0.81; 95% CI = 0.52–1.25) (Tables 2 and 3).

**Table 2:** Distribution of CYP1A1 rs4646903 among laryngeal cancer cases and controls included in this meta‐analysis

First author‐year	Ethnicity (country of origin)	Total sample (case/control)	Source of control	Laryngeal cancer			Controls		
				AA	AG	GG	AA	AG	GG
Park‐1997	Mixed (USA)	23/23	HB	18	5^a^		21	2^a^	
Morita‐1999	Asian (China)	69/164	PB	48	16	5	104	54	6
Varzim‐2003	Caucasian (Portugal)	88/178	PB	68	19	1	135	43	0
Li‐2004	Asian (China)	89/164	HB	33	24	32	77	60	27
Sam‐2008	Asian (India)	80/220	HB	64	15	1	180	36	4
Sharma‐2010	Asian (India)	46/201	HB	23	13	10	156	34	11
Szanyi‐2012	Caucasian (Hungary)	48/150	HB	34	14	0	119	31	0

a The number of the combined AG and GG genotypes.

PB: population based; HB: hospital based. AA indicates wild‐type, AG indicates heterozygote, GG indicates variant homozygote.

**Table 3:** Summary ORs for various contrasts of CYP1A1 rs1048943 and rs4646903 polymorphisms in this meta‐analysis

	Number of studies	(AG + GG) *versus* AA	GG *versus* AA
		OR (95% CI) *P* (*Q*‐test)	OR (95% CI) *P* (*Q*‐test)
rs1048943			
Total	6	1.86 (1.45–2.40) 0.000	1.77 (1.28–2.81) 0.007
Caucasian	2	0.81 (0.52–1.25) 0.217	0.93 (0.49–1.39) 0.293
Asian	3	3.90 (2.70–5.64) 0.000	3.29 (1.88–4.49) 0.006
rs4646903			
Total	7	1.33 (1.04–1.71) 0.029	1.53 (1.31–2.21) 0.012
Caucasian	2	1.14 (0.71–1.81) 0.269	1.36 (0.79–2.31) 0.193
Asian	4	1.39 (1.03–1.87) 0.009	1.52 (1.25–2.32) 0.002

*P* (*Q*‐test): *P*‐value of *Q*‐test for heterogeneity test; OR: odds ratio; CI: confidence interval.

**Figure 3:**
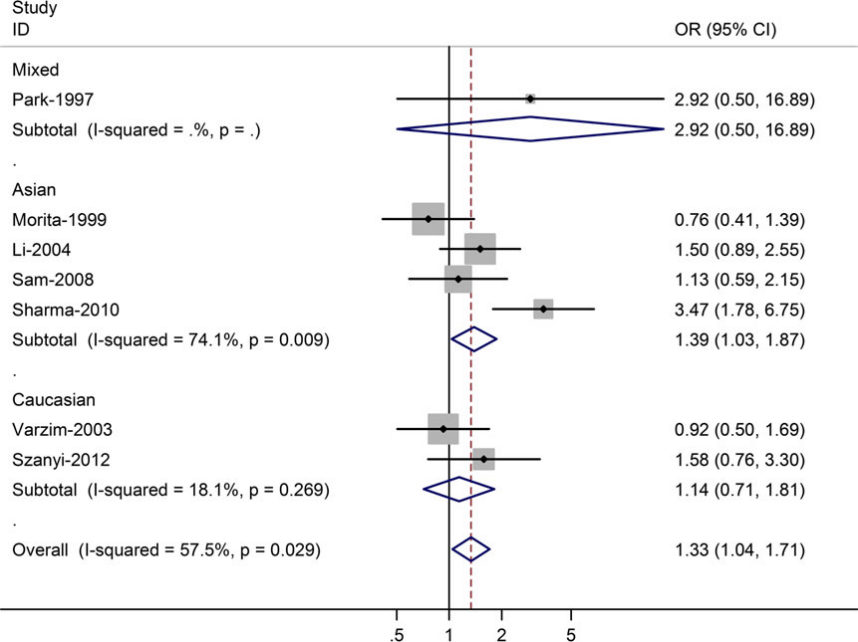
Forest plot (random‐effects model) of laryngeal cancer risk associated with CYP1A1 rs4646903 polymorphism for (AG + GG) *versus* AA.

The association between *CYP1A1* rs4646903 and laryngeal cancer risk was performed by meta‐analysis. Overall, for the homozygote G/G and G allele carriers (A/G + G/G), the pooled ORs for seven studies were 1.53 (95% CI = 1.31–2.21, *P* = 0.012 for heterogeneity) and 1.33 (95% CI = 1.04–1.71, *P* = 0.029 for heterogeneity) (Fig. 3), when compared with the homozygous wild‐type genotype (A/A). In the stratified analysis by ethnicity, significant risks were found among Asians for both the G allele carriers (OR = 1.39, 95% CI = 1.03–1.87; *P* = 0.009 for heterogeneity) and homozygote G/G (OR = 1.52; 95% CI = 1.25–2.32; *P* = 0.002 for heterogeneity). Among Caucasian populations, no significant association was found in G/G *versus* A/A (OR = 1.14; 95% CI = 0.71–1.81), and G allele carriers *versus* A/A (OR = 1.36; 95% CI = 0.79–2.31).

### Sensitivity analyses

A single study involved in the meta‐analysis was deleted each time to reflect the influence of the individual data set to the pooled ORs, and the corresponding pooled ORs were not materially altered (data not shown).

### Publication bias

**Figure 4:**
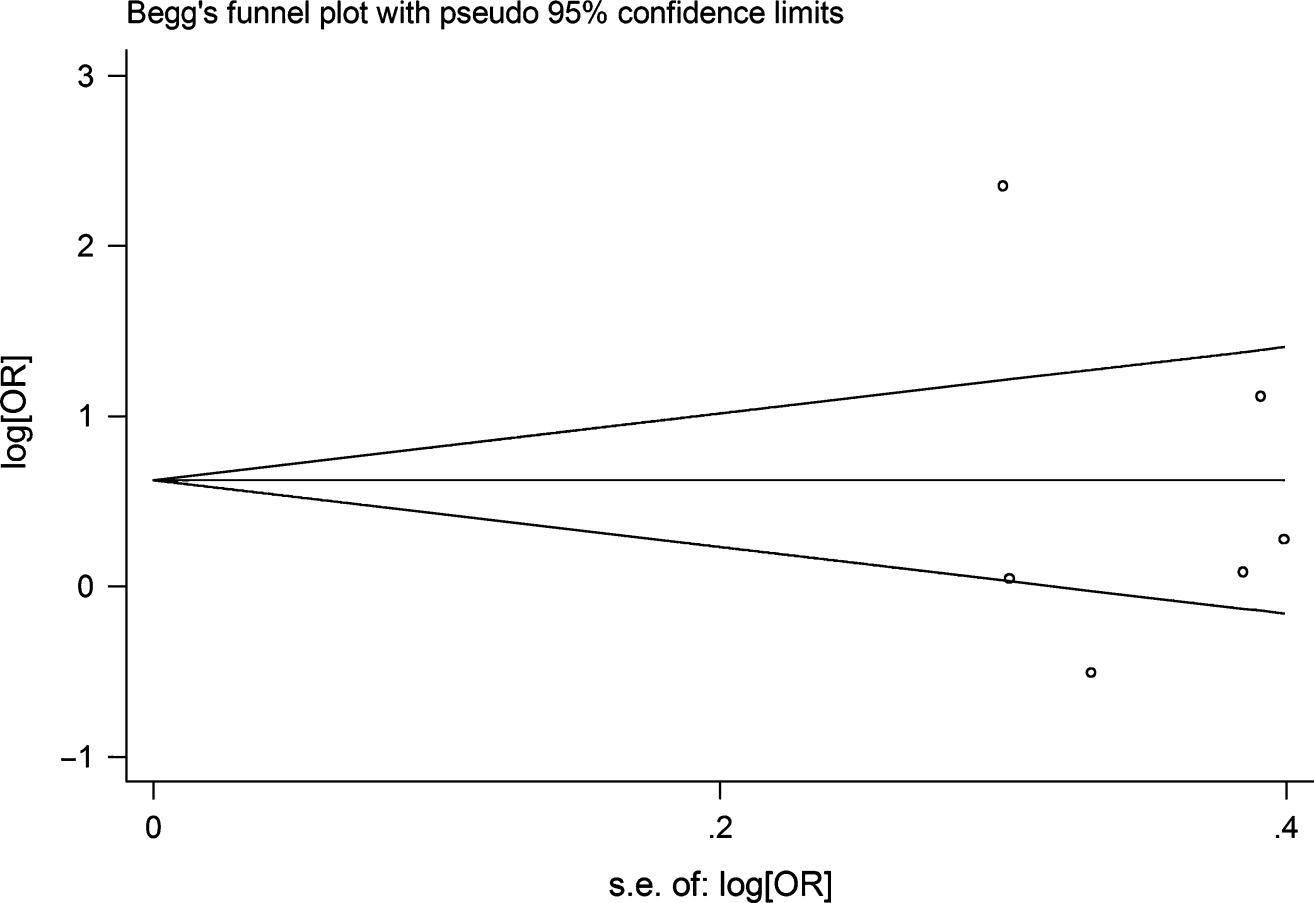
Begg's funnel plot of CYP1A1 rs1048943 polymorphism and laryngeal cancer risk for (AG + GG) *versus* AA.

**Figure 5:**
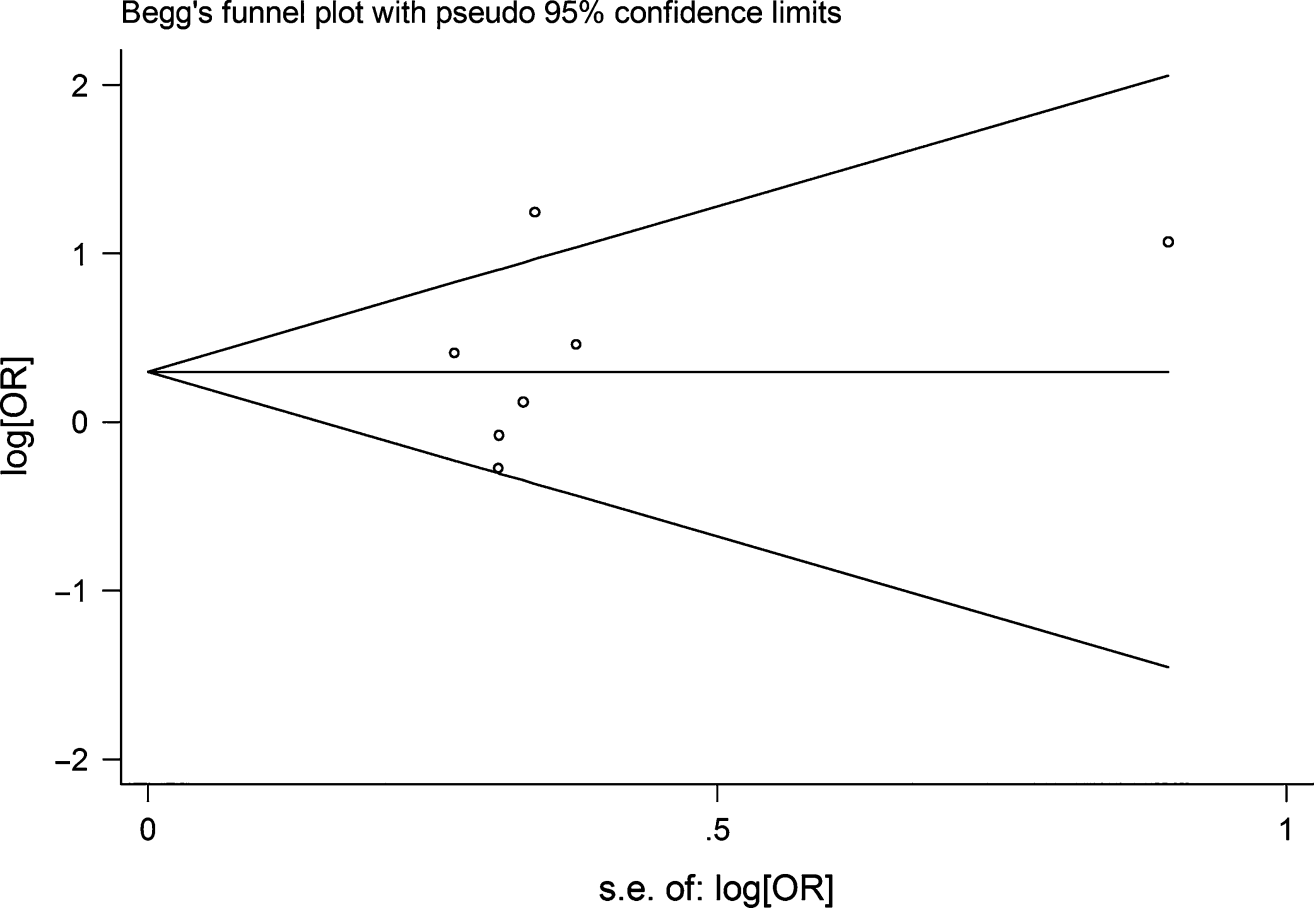
Begg's funnel plot of CYP1A1 rs4646903 polymorphism and laryngeal cancer risk for (AG + GG) *versus* AA.

Begg's funnel plot and Egger's test were performed to access the publication bias of literatures. Evaluation of publication bias for G allele carriers *versus* AA between *CYP1A1* rs1048943 and laryngeal cancer risk showed that the Egger test was not significant (*P* = 0.615). The funnel plots for publication bias (Fig. 4) also did not show some asymmetry. The similar results were found between *CYP1A1* rs4646903 and laryngeal cancer risk for G allele carriers *versus* AA (Fig. 5). These results did not indicate a potential for publication bias.

## Discussion

CYP genes which are comprised of large families of endoplasmic and cytosolic enzymes play a role in drug, steroid hormone and pro‐carcinogen metabolism. In humans, the CYPP450s complex (metalloproteins) contains more than 15 different enzymes 23. Some CYP haeme‐thiolate enzymes participate in the detoxification and formation of reactive intermediates of thousands of chemicals that can damage DNA, lipids and proteins. *CYP1A1* is a critical CYPP450 and studies suggest that a *CYP1A1* polymorphism may be a risk factor for several malignancies even in the face of its role in detoxification of environmental carcinogens and metabolic activation of dietary compounds that protect against cancer. Two functional non‐synonymous polymorphisms have been recently studied. Specifically, a T‐to‐C mutation in the non‐coding 3′‐flanking region has been reported to cause the creation a new MspI restriction site (MspI polymorphism m1, T6235C, rs4646903). Another *CYP1A1* polymorphism is a G‐to‐A transition (A4889G) in exon 7, resulting in the replacement of isoleucine (Ile) by valine (Val), which is a haeme‐binding site (Ile462Val polymorphism m2, A4889G, rs1048943) 24.

It has been accepted that polymorphism of genes involved in the metabolism of tobacco carcinogen is candidate loci for laryngeal cancer susceptibility. Accumulating studies regarding gene variants in the carcinogen metabolism pathway have been performed. It is well recognized that there is a range of individual susceptibility to the same kind of cancer even with identical environmental exposure. Host factors, including polymorphisms of genes involved in carcinogenesis, may have accounted for this difference. Therefore, genetic susceptibility to cancer has been a research focus in scientific community.

Previous meta‐analyses suggest that genetic variation of CYP1A1 rs1048943 and rs4646903 might be associated with increased risks of various cancers, such as lung cancer 25 and oesophageal cancer 26. However, no significant association of CYP1A1 rs1048943 and rs4646903 polymorphisms with increased susceptibility to breast cancer has been reported by a recent meta‐analysis 27. The present meta‐analysis investigating the relationship between *CYP1A1* rs1048943 and rs4646903 polymorphisms and laryngeal cancer risk provided the most comprehensive evidence. Significant association of *CYP1A1* rs1048943 and rs4646903 polymorphisms and laryngeal cancer risk was demonstrated. Meta‐analyses of total studies showed that *CYP1A1* rs1048943 and rs4646903 polymorphisms were associated with laryngeal cancer risk for the homozygote G/G and G allele carriers (A/G + G/G) when compared with the homozygous wild‐type genotype (A/A). In the stratified analysis by ethnicity, the significantly risks were found among Asians for both the G allele carriers and homozygote G/G. However, no significant associations were found in Caucasian population all genetic models. Sensitivity analyses by sequential omission of any individual studies and subgroup analyses by case sample size and ethnicity suggested the findings were highly unlikely because of chance. In the stratified analysis by ethnicity, the significantly risks were found among Asians, but no among Caucasians. Publication bias analysis demonstrated that bias favouring publications of positive studies was inexistent. Meanwhile, because the same polymorphism seemed to play different roles in *CYP1A1* rs1048943 and rs4646903 susceptibility among different ethnic populations and because the frequencies of SNPs were different among different ethnic groups, subgroup analyses based on ethnicity were conducted.

Our data were consistent with the results of a previous meta‐analysis 28 included only seven studies published in 2009. We have improved upon that previous meta‐analysis by including more recent related studies and by generally using a more comprehensive search strategy. Screening, study selection and quality assessment were performed independently and reproducibly by two reviewers. We also explored heterogeneity and potential publication bias in accordance with published guidelines.

In statistics, meta‐analysis comprises statistical methods for contrasting and combining results from different studies in the hope of identifying patterns among study results, sources of disagreement among those results or other interesting relationships that may come to light in the context of multiple studies. However, some limitations of this meta‐analysis should be acknowledged. Firstly, heterogeneity is a potential problem when interpreting all the results of meta‐analyses. Although we minimized the likelihood by performing a careful search for published studies, using the explicit criteria for study inclusion, performing data extraction and data analysis strictly, the significant between‐study heterogeneity still existed in almost each comparison. The presence of heterogeneity can result from differences in the selection of controls, age distribution, lifestyle factors and so on. Although most of the controls were selected from healthy populations, some studies had selected controls among friends or family of laryngeal cancer patients or patients with other diseases. Secondly, only published studies were included in this meta‐analysis. The presence of publication bias indicates that non‐significant or negative findings may be unpublished. Lastly, our results were based on unadjusted estimates, while a more precise analysis should be conducted if individual data were available, which would allow for the adjustment by other covariates including age, ethnicity, family history, environmental factors and lifestyle.

In conclusion, this meta‐analysis suggests that the *CYP1A1* rs1048943 and rs4646903 polymorphisms are associated with laryngeal cancer risk among Asian populations. In addition, it is necessary to conduct large trials using standardized unbiased methods, homogeneous laryngeal cancer patients and well‐matched controls, with the assessors blinded to the data.

## Conflicts of interest

The authors confirm that there are no conflicts of interest.
